# Cryo-Electron Microscopy of *Arabidopsis thaliana* Phytochrome A in Its Pr State Reveals Head-to-Head Homodimeric Architecture

**DOI:** 10.3389/fpls.2021.663751

**Published:** 2021-04-21

**Authors:** Weixiao Yuan Wahlgren, David Golonka, Sebastian Westenhoff, Andreas Möglich

**Affiliations:** ^1^ Department of Chemistry and Molecular Biology, University of Gothenburg, Gothenburg, Sweden; ^2^ Lehrstuhl fur Biochemie, Universität Bayreuth, Bayreuth, Germany; ^3^ Bayreuth Center for Biochemistry and Molecular Biology, Universität Bayreuth, Bayreuth, Germany; ^4^ North-Bavarian NMR Center, Universität Bayreuth, Bayreuth, Germany

**Keywords:** single particle, signal transduction, sensory photoreceptor, phytochrome, cryo-electron microscopy

## Abstract

Phytochrome photoreceptors regulate vital adaptations of plant development, growth, and physiology depending on the ratio of red and far-red light. The light-triggered *Z*/*E* isomerization of a covalently bound bilin chromophore underlies phytochrome photoconversion between the red-absorbing Pr and far-red-absorbing Pfr states. Compared to bacterial phytochromes, the molecular mechanisms of signal propagation to the C-terminal module and its regulation are little understood in plant phytochromes, not least owing to a dearth of structural information. To address this deficit, we studied the *Arabidopsis thaliana* phytochrome A (*At*phyA) at full length by cryo-electron microscopy (cryo-EM). Following heterologous expression in *Escherichia coli*, we optimized the solvent conditions to overcome protein aggregation and thus obtained photochemically active, near-homogenous *At*phyA. We prepared grids for cryo-EM analysis of *At*phyA in its Pr state and conducted single-particle analysis. The resulting two-dimensional class averages and the three-dimensional electron density map at 17 Å showed a homodimeric head-to-head assembly of *At*phyA. Docking of domain structures into the electron density revealed a separation of the *At*phyA homodimer at the junction of its photosensor and effector modules, as reflected in a large void in the middle of map. The overall architecture of *At*phyA resembled that of bacterial phytochromes, thus hinting at commonalities in signal transduction and mechanism between these receptors. Our work paves the way toward future studies of the structure, light response, and interactions of full-length phytochromes by cryo-EM.

## Introduction

Identified first among the plant sensory photoreceptors (Butler et al., 1959), phytochromes (phy) serve as ratiometric sensors of red and far-red (i.e., near-infrared) light and coordinate a wide range of vital adaptations of physiology, for instance shade avoidance, morphogenesis, development, and the timing of germination and flowering (Casal, 2013; Hughes, 2013; Pham et al., 2018; Legris et al., 2019; Rockwell and Lagarias, 2020). Land plants express a varying number of phys, with the model organism *Arabidopsis thaliana* possessing five, denoted *At*phyA through *At*phyE. Owing to their abundance and predominant role in photomorphogenesis, *At*phyA and *At*phyB have been studied more extensively than *At*phyC-*At*phyE (Legris et al., 2019). Following their initial discovery in plants, phys were also identified in fungi and bacteria, thus providing experimentally tractable systems for the study of photochemistry, structure, and signal transduction.

**Figure 1:**
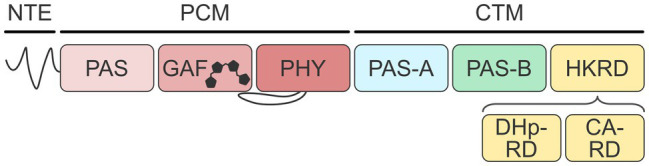
Architecture of plant phytochromes. Phytochromes from land plants possess a photosensory core module (PCM) and a C-terminal module (CTM). The PCM consists of an N-terminal extension, a PAS domain, a bilin-binding GAF domain, and a PHY domain. The CTM contains additional PAS-A, PAS-B, and histidine-kinase-related domains (HKRD). The HKRD in turn comprises DHp-RD and CA-RD subdomains.

Phytochromes generally have bipartite composition with an N-terminal photosensory core module (PCM, alternatively also called photosensory module, PSM) and a C-terminal module (CTM). As exemplified for *At*phyA (Figure 1), the PCM comprises an N-terminal extension (NTE) and concatenated PAS (Per-ARNT-Sim; Möglich et al., 2009), GAF (cyclic GMP, adenylyl cyclase, FhlA; Aravind and Ponting, 1997), and PHY (phytochrome-specific) domains; notably, the NTE is absent from bacterial phys (BphP; Rockwell and Lagarias, 2020). A linear tetrapyrrole (i.e., bilin) chromophore is nestled within the GAF domain and covalently attached to a cysteine residue residing inside the GAF domain itself in case of land plants, or within the PAS module in case of bacterial and fungal phys. As a chromophore, plant phytochromes naturally use phytochromobilin (P*Φ*B), a reduced derivative of biliverdin, but can also accommodate the cyanobacterial phycocyanobilin (PCB) without loss of photosensitivity (Kami et al., 2004). In darkness, phys of land plants generally adopt their red-absorbing Pr state which is characterized by the bilin chromophore in the *Z* configuration of its C15〓C16 double bond (see Figure 1b of Golonka et al., 2019; Legris et al., 2019). Light absorption drives the conversion to the metastable light-adapted Pfr state with the bilin in the 15*E* configuration. The Pfr state reverts to the Pr state thermally or upon illumination with far-red light.

The CTM of plant phys consists of two additional PAS domains, denoted PAS-A and PAS-B, and a histidine-kinase-related domain (HKRD) which, however, misses key residues and consequently lacks histidine kinase activity. The HKRD can be further subdivided into two subdomains, denoted DHp-related (DHp-RD) and CA-related domains (CA-RD), based on their homology to the dimerization and phospho-accepting histidine (DHp) and catalytic (CA) domains of bacterial two-component systems (Gao and Stock, 2009). The PCM and CTM together form versatile signaling hubs that process light signals and, at least in case of *At*phyB, temperature cues (Legris et al., 2016; Casal and Balasubramanian, 2019). A cohort of physiological responses is elicited *via* light-dependent protein:protein interactions, nucleocytoplasmic shuttling, proteolytic degradation, and potentially serine/threonine kinase activity (Yeh and Lagarias, 1998). Although the isolated PCMs of *At*phyA and *At*phyB are capable of entering stringently light-regulated interactions with partner proteins (Levskaya et al., 2009; Toettcher et al., 2011; Pham et al., 2018; Golonka et al., 2019, 2020), in particular the phytochrome-interacting factors, many other output modes strictly require the presence of the CTM (Legris et al., 2019). Likewise, the CTM contributes to stabilizing the dimeric structure of plant phys (Legris et al., 2019).

Pioneering studies on bacterial receptors have provided molecular insight into phy structure and signal transduction. Following the elucidation of the truncated PAS-GAF PCM fragment (Wagner et al., 2005), the complete PAS-GAF-PHY PCM of several bacterial phys was structurally resolved in both the Pr and Pfr states (Essen et al., 2008; Yang et al., 2008; Takala et al., 2014; Burgie et al., 2016). A particular detailed view was achieved for the homodimeric model BphP from *Deinococcus radiodurans* (*Dr*BphP) by integrating X-ray crystallography, solution scattering, and molecular dynamics (Takala et al., 2014). Light absorption by the dark-adapted *Dr*BphP drives the 15*Z*/*E* isomerization of the chromophore which induces a protrusion of the PHY domain, denoted PHY tongue, to change conformation from β hairpin to α helix. As a result, the distance between the GAF and PHY domains diminishes, and within the homodimeric assembly the PHY subunits splay apart (Takala et al., 2014, 2020). Comparative studies on a range of BphPs suggest that the principal structural response is widely shared across phys, at least those of bacterial origin (Björling et al., 2015). A concomitant structural change of a long helix, which connects the PHY and PAS/GAF domains, has been observed by nuclear magnetic resonance spectroscopy (Isaksson et al., 2020) and may result in asymmetric modification of the dimeric phy assembly upon photoactivation (Gourinchas et al., 2018). A handful of BphPs were also atomically resolved as intact receptors with associated CTMs (Bellini and Papiz, 2012; Otero et al., 2016; Gourinchas et al., 2017; Etzl et al., 2018). With but few exceptions (Bellini and Papiz, 2012; Otero et al., 2021), the full-length BphPs invariably adopted a parallel homodimeric assembly which places the PCM and CTM in tandem, connected *via* a homodimeric α-helical coiled coil. Although BphP-histidine kinases, as the most frequent BphP class, have to date eluded structural elucidation at atomic resolution, cryo-electron microscopy and solution scattering suggest that they also conform to this principal arrangement (Li et al., 2010; Björling et al., 2016). Solution scattering indicates that light signals are transduced from the BphP PCM to the histidine kinase through the coiled coil as rotary rearrangements, similar to other histidine kinases (Björling et al., 2016; Ohlendorf et al., 2016; Berntsson et al., 2017; Buschiazzo and Trajtenberg, 2019; Möglich, 2019; Takala et al., 2020).

By contrast, the structural characterization of plant phys lags that of the bacterial systems, not least owing to the more challenging preparation of proteins in sufficient quantity and quality. High-resolution information is to date available for the PCM of plant phys but not for the CTM, let alone full-length proteins. The crystal structure of the PAS-GAF-PHY PCM (*sans* the NTE) of *At*phyB overall resembled that of the bacterial phys, excepting the orientation and length of several protein loops (Burgie et al., 2014). Only recently, the PAS-GAF and PAS-GAF-PHY structures of two plant phyB proteins and the first PAS-GAF structure of plant phyA were reported (Nagano et al., 2020). Notably, the individual phyA and phyB fragments exhibited similar structures, despite distinct physiological roles associated with these phytochromes, at least in *A. thaliana* (Legris et al., 2019). Structural data on plant phys at full length are scarce and, where available, only of low resolution. Negative-staining electron microscopy and X-ray solution scattering suggested that the homodimeric phyA from *Pisum sativum* comprises four lobes arranged in a cruciform shape (Nakasako et al., 1990, 2005; Oide and Nakasako, 2021). Recently, similar studies were performed on full-length phyB from *A. thaliana* (Oide et al., 2020). Based again on electron microscopy and solution scattering, a cruciform assembly was also advanced for the homodimeric *At*phyB, overall similar to the shape proposed for *P. sativum* phyA (Oide and Nakasako, 2021). Based on the crystal structure of the *At*phyB PCM (Burgie et al., 2014), the structural models predicted that in the homodimeric phyA and phyB the PAS and GAF domains of the two PCM copies are splayed apart and thus separated (Oide et al., 2020; Oide and Nakasako, 2021). Similarly, the models posited that the two CTMs are likewise tilted apart. Interestingly, these findings are inconsistent with another study by electron microscopy which proposed a head-to-head dimeric arrangement for full-length *At*phyB (Burgie et al., 2017). Notably, this head-to-head assembly is compatible with the high-resolution structures of isolated plant phy PCMs known to date, see above, which invariably revealed tight head-to-head association between the two PAS and GAF domains from each subunit of the dimeric molecule.

To expand our knowledge of the structure and mechanism of plant phytochromes, we here investigated *A. thaliana* phyA by cryo-electron microscopy. To this end, we established protocols for the heterologous expression of *At*phyA in *Escherichia coli* and its near-homogenous preparation. We thus identified solution conditions at which the inherent aggregation of *At*phyA is greatly alleviated, yet intact photochemical response preserved. Analysis by cryo-electron microscopy and single-particle averaging yielded a structural model at ~17 Å resolution and revealed a parallel homodimeric architecture for *At*phyA with the two PCM copies in tight complex rather than splayed apart.

## Materials and Methods

### Heterologous Expression and Purification of *At*phyA

The gene encoding full-length *At*phyA (Unicode entry PHYA_ARATH, residues 1-1122) was obtained from M.D. Zurbriggen and subcloned onto the pCDF backbone (Novagen) using PCR amplification and Gibson cloning (Gibson et al., 2009). The gene was thus equipped with a C-terminal hexahistidine tag and placed under control of a lactose-inducible T7 promoter. As in a previous study (Golonka et al., 2019), the resultant plasmid pDG388 also harbored a second expression cassette under lactose-inducible T7 control that comprised the genes for *Synechocystis* sp. heme oxygenase and the biliverdin reductase PcyA, originally subcloned from the pKT270 plasmid (Mukougawa et al., 2006). A plasmid map of pDG388 is supplied as Supplementary Material. The plasmid pDG388 was confirmed by Sanger DNA sequencing (Microsynth) and transformed into *E. coli* BL21(DE3) LOBSTR cells (Andersen et al., 2013). Transformed bacteria were grown at 37°C and 225 rpm in 8 × 1,000 ml TB (terrific broth) medium supplemented with 100 μg ml^−1^ streptomycin. Once the optical density at 600 nm reached around 0.6, the incubation temperature was lowered to 18°C, 0.5 mM δ-aminolevulinic acid was added, and expression was induced by 1 mM isopropyl-β-thiogalactopyranoside. Following incubation at 18°C for 2 days, cells were harvested by centrifugation, resuspended in 50 mM Tris/HCl pH 8.0, 20 mM NaCl, 20 mM imidazole, and lysed by ultrasound. After clearing by centrifugation, the suspension was applied to a 1-ml Protino Ni-NTA column (Macherey-Nagel) using an Äkta prime chromatography system. Protein was eluted using a gradient from 2 to 500 mM imidazole over 50 ml. Fractions of 1 ml were collected and analyzed by denaturing polyacrylamide gel electrophoresis. Covalently bound chromophore and total protein were visualized by Zn-induced fluorescence (Berkelman and Lagarias, 1986) and Coomassie staining, respectively. Fractions were pooled based on purity and dialyzed into 50 mM Tris/HCl pH 8.0, 20 mM NaCl, 10% (w/v) glycerol. Samples were concentrated by spin filtration (Amicon Ultra spin column, molecular-weight cutoff 10,000 Da), flash-frozen in liquid nitrogen and stored at −80°C. In the course of optimizing solution conditions, *At*phyA was purified at different pH values. To this end, all solutions were buffered with 50 mM sodium carbonate at pH 9.3 or pH 10.6, respectively.

### UV/Vis Spectroscopy

Absorbance spectroscopy was performed on an Agilent 8453 diode-array spectrophotometer at 22°C. To determine the concentration of *At*phyA, a molar extinction coefficient of 21,200 M^−1^ cm^−1^ at 672 nm was used. Absorbance spectra were recorded for dark-adapted *At*phyA and after saturating illumination with LEDs of (650 ± 15) nm and (720 ± 15) nm emission wavelength. The Pr:Pfr ratio obtained after 650-nm illumination was calculated according to (Butler et al., 1964).

### Size-Exclusion Chromatography

Prior to cryo-electron microscopy experiments, the frozen *At*phyA samples were thawed and purified by size-exclusion chromatography. Size-exclusion chromatography (SEC) of full-length *At*phyA was performed at 12°C using an Äkta system (GE Healthcare) with a flow rate of 0.3 ml min^−1^. A Superose 6 10/300 GL column (GE Healthcare) was equilibrated in 20 mM Tris/HCl pH 8.0, or 50 mM sodium carbonate buffer pH 9.3, respectively. Prior to loading, *At*phyA samples were converted to Pr state by illuminating with 730 nm far-red light.

### Single-Particle cryo-EM Grid Preparation and Data Collection

Cryo-EM grids were prepared under dim green safe light using a Vitrobot (FEI) with the sample chamber at 4°C and 100% humidity. Three microliter protein sample (1–1.5 mg ml^−1^) was pre-illuminated with 730 nm far-red light and applied to glow-discharged C-flat Holey carbon R 2/2-300 grids. Excess solution was removed by blotting filter paper, and the grids were plunge-frozen in liquid ethane. The samples were imaged using a Titan Krios operated at 300 kV and a magnification of 130,000-fold. The images were recorded on a Quantum K2 camera with pixel size of 1.06 Å and an exposure rate of 70 electrons per Å^2^ for a total 40 frames. The targeted defocus range was varied from −1 to −2.5 μm using the EPU software (ThermoFisher).

### Single Particle cryo-EM Data Processing

A total of 1,221 movies were collected, motion-corrected, and contrast transfer function (CTF)-estimated using cryoSPARC2 (Punjani et al., 2017). After the micrographs were denoised using Topaz denoise, a small number of particles (~450) were manually picked and subjected to two-dimensional classification to generate references for the template picker in cryoSPARC2 (Bepler et al., 2020). About 271,000 particles were initially autopicked and extracted. Junk particles were removed by several rounds of 2D class averaging, and the good particles were used in Topaz deep pick (Bepler et al., 2019). Particles were further cleaned up with two more rounds of 2D class averaging. A set of 136,173 particles was used to reconstruct an initial model using Ab-initio in cryoSPARC2. The model was further refined using homogeneous refinement with 2-fold symmetry. The final model has a resolution of approximately 17 Å based on gold-standard Fourier shell correlation at 0.143 cutoff (Scheres and Chen, 2012).

### Docking of Crystal Structures Into the Electron Density Map

*At*phyA model analysis and crystal-structure docking to the low-resolution model were performed in UCSF Chimera (Pettersen et al., 2004). The crystal structures of the PAS-GAF domains of phyA from *Glycine max* (PDB code 6TC7; Nagano et al., 2020), the PHY domain of plant phyB from *A. thaliana* (4OUR; Burgie et al., 2014), the PAS domain of the BphP from *Xanthomonas campestris* (5AKP; Otero et al., 2016), and the PAS-linked histidine kinase from *Thermotoga maritima* (3A0R; Yamada et al., 2009) were used for the docking.

## Results

### Preparation of Full-Length *At*phyA

**Figure 2:**
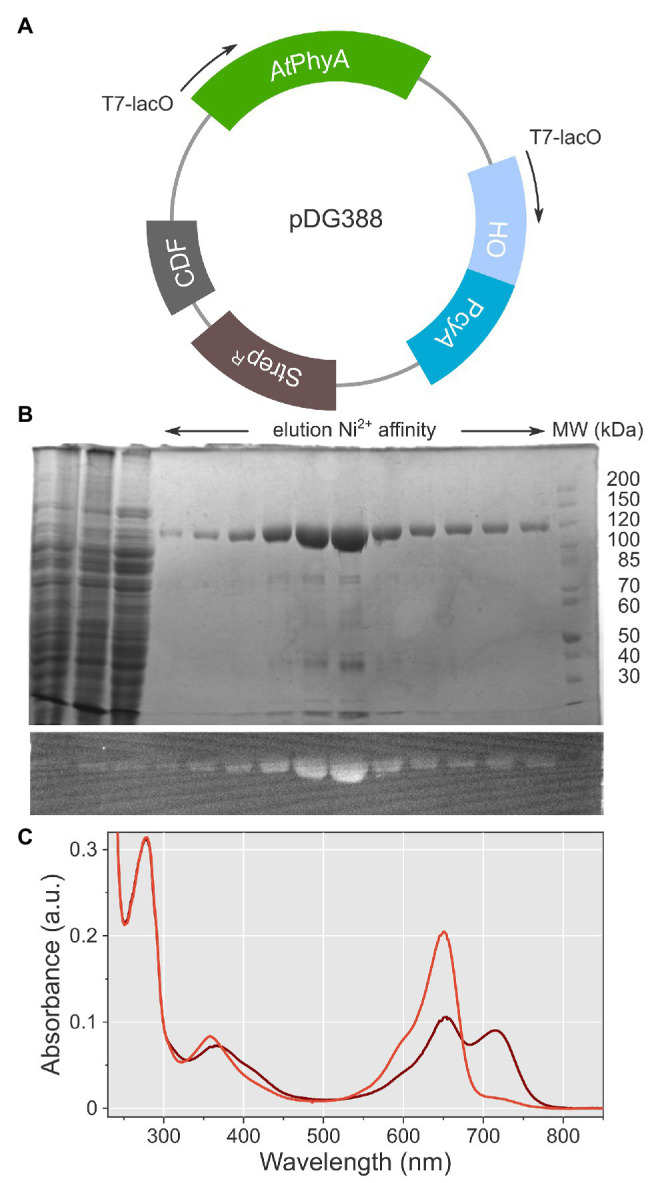
Expression, purification, and spectroscopic characterization of full-length *At*phyA. **(A)** The plasmid pDG388 harbors expression cassettes for *At*phyA and for heme oxygenase (HO)/PcyA, respectively. Both cassettes are expressed from T7 promoters under the control of *lacO*. The vector carries a CDF origin of replication and a streptomycin resistance marker (StrepR). **(B)** Purification of *At*phyA by Ni^2+^ immobilized metal ion affinity chromatography. The upper panel shows a Coomassie-stained denaturing polyacrylamide gel, and the lower panel the corresponding zinc-induced bilin fluorescence. **(C)** UV/vis absorbance spectra of *At*phyA after illumination with 650 nm (brown) and 720 nm (red), respectively.

To facilitate biophysical analyses, especially structural investigation, we set out to establish an efficient protocol for the heterologous expression in *E. coli* of *At*phyA at full length. To this end, we adapted a pCDF plasmid system previously employed for the production of the *At*phyB PCM at good yield and purity (Golonka et al., 2019, 2020). By embedding the *At*phyA gene in this system, it was furnished with a C-terminal hexahistidine affinity tag and placed under control of a T7-lacO promoter (Figure 2A). To supply the phycocyanobilin chromophore during heterologous expression, the plasmid additionally bore a T7-lacO-controlled bicistronic operon which encodes the *Synechocystis* sp. heme oxygenase (HO) and the biliverdin reductase PcyA (Mukougawa et al., 2006). The plasmid was transformed into the *E. coli* LOBSTR expression strain (Andersen et al., 2013), a derivative of BL21 that carries the DE3 lysogen and genomic modifications that alleviate the contamination by endogenous bacterial proteins during purification by immobilized metal-ion affinity chromatography (IMAC). Protein expression was performed over 2 days at 18°C. To improve chromophore supply during expression, 0.5 mM δ-aminolevulinic acid was added as a precursor in the biosynthesis of porphyrin (Shemin and Russell, 1953), from which in turn biliverdin and other bilins derive. Even after a single purification step by IMAC, full-length *At*phyA could be obtained with covalently attached PCB, as detected by zinc-induced bilin fluorescence (Berkelman and Lagarias, 1986), and but few contaminants, as revealed by Coomassie staining (Figure 2B). Per liter of bacterial culture, around 1 mg *At*phyA could routinely be prepared.

We used UV/vis absorbance spectroscopy to assess the photochemical integrity of the protein preparation (Figure 2C). In its dark-adapted state, *At*phyA adopted a mixed population of the Pr and Pfr states, with Q-band absorbance maxima at 650 and 715 nm, respectively. Illumination with red light (650 nm) achieved a Pfr:Pr ratio of 0.57:0.43. Under far-red light (720 nm), *At*phyA was converted almost entirely to its Pr state with little Pfr remaining. In the Pr state, the absorbance ratio of the Q band and the Soret band at 358 nm amounted to 2.4.

### Optimization of Solution Conditions for Structural Analysis

**Figure 3:**
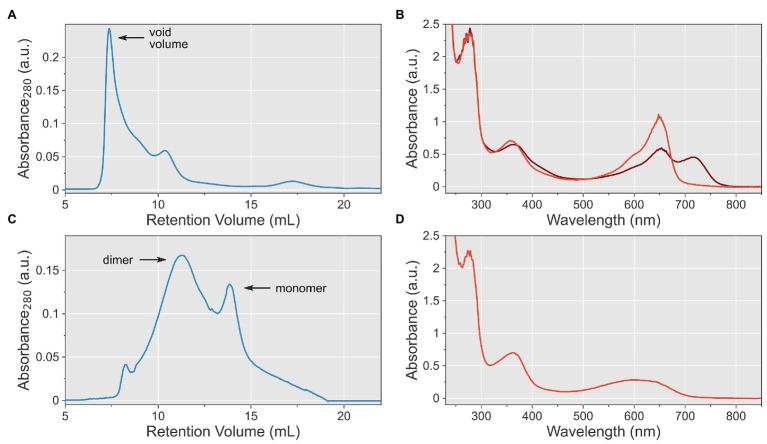
Optimization of solution conditions for purification of *At*phyA at full length. **(A)** Size-exclusion chromatography (SEC) of *At*phyA at pH 8.0. The void volume of the column is indicated. **(B)** UV/vis absorbance spectra of *At*phyA at pH 9.3 after illumination with 650 nm (brown) and 720 nm (red), respectively. **(C)** SEC of *At*phyA at pH 9.3 reveals a mixture of apparent dimeric and monomeric species. **(D)** UV/vis absorbance spectrum of *At*phyA at pH 10.6.

Having ascertained intact photochemistry, we next used size-exclusion chromatography for further purification and to assess the solution properties and homogeneity of the *At*phyA sample. The chromatographic analysis revealed higher-order *At*phyA aggregates that eluted within the void volume of the column (Figure 3A). Based on the molecular weight range the column can resolve, we estimated a size of these aggregates in the mega-Dalton range. Attempts at dissolution of the *At*phyA aggregates by dialysis into different buffers failed.

Reasoning that the aggregates formed irreversibly, we set out to alter the pH value at which protein purification was conducted. Given an isoelectric point (pI) for *At*phyA of 5.9, we opted for higher pH values than 8.0 in the initial attempt. At pH 9.3, *At*phyA could be purified by IMAC with similar yield and purity as before. Absorbance spectroscopy revealed that the Q-band intensity somewhat decreased relative to that of the Soret band (ratio 1.5:1; Figure 3B). A similar attenuation of the Q band at alkaline pH was for instance reported for both bacterial phytochromes and cyanobacteriochromes and was ascribed to partial deprotonation of the bilin chromophore (Hirose et al., 2013; Rumfeldt et al., 2019). That notwithstanding, *At*phyA showed intact and reversible Pr↔Pfr photoconversion at this pH value. Using SEC, we went on to assess the oligomeric state of the protein preparation in solution and found it markedly improved (Figure 3C). In contrast to the SEC run at the lower pH which was dominated by high-weight aggregates, the run at pH 9.3 revealed a mixture of *At*phyA dimers and monomers with but few larger aggregates. To assess whether the solution properties could be further enhanced, we elevated the pH to 10.6. Following expression and purification, the UV/vis spectroscopic analysis, however, showed an altered Q-band absorption without fine structure, of lower intensity and shifted to shorter wavelengths (Figure 3D). Given that this spectrum resembled that of isolated PCB, we concluded that at this pH chromophore binding is severely perturbed although covalent attachment to the *At*phyA protein evidently still took place during expression in *E. coli*. Illumination with red light did not elicit any spectral changes, and we hence discontinued the experiments at a pH of 10.6.

### Structural Analysis of Full-Length *At*phyA by cryo-Electron Microscopy

**Figure 4:**
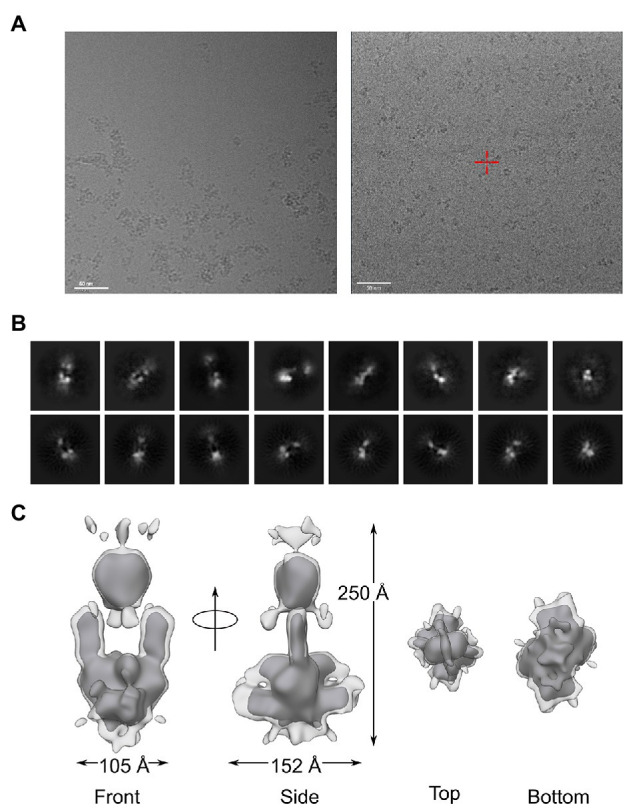
Structural analysis of full-length *At*phyA by single particle cryo-electron microscopy. **(A)** Electron micrographs for *At*phyA prepared at pH 8.0 (left) or pH 9.3 (right). The scale bar indicates a length of 50 nm. **(B)** Selected reference-free 2D classes of *At*phyA (top row) in comparison with the reprojections of the 3D cryo-EM model (bottom row). **(C)** The 3D electron density map at ~17 Å resolution demonstrates a head-to-head dimeric arrangement of *At*phyA. The light and dark gray densities are contoured at different levels, see main text.

We next prepared grids for the structural analysis of full-length *At*phyA by cryo-EM. To this end, *At*phyA was first illuminated with 730 nm to convert it to its Pr state. Samples were then applied to carbon grids and plunge-frozen. When initially *At*phyA was prepared at pH 8.0, the electron micrographs showed pervasive protein aggregation, despite prior filtration of the sample (Figure 4A). These observations concurred with the SEC analysis (see Figure 3A) and effectively precluded further structural analysis at this pH. By contrast, grids prepared for *At*phyA purified at pH 9.3 yielded dispersed particles (Figure 4A), again consistent with SEC (see Figure 3C), and were used for subsequent analysis. Following Topaz denoising, single particles were picked manually and based on templates. We tested different box sizes to extract the picked particles, with the best 2D class averages obtained for a box size of 448 pixels (Figure 4B). From 136,173 particles, the reference-free initial model of *At*phyA was acquired without imposing any symmetry. After refinement with 2-fold symmetry imposed, a final 3D electron density map of *At*phyA was obtained with a resolution of approximately 17 Å (Figure 4C). Two-dimensional projections of the map resembled the class averages of the experimental data (Figure 4B).

The map revealed an oblong shape with approximately 2-fold symmetry and dimensions of around 100 Å × 150 Å × 250 Å. The size and the form of the map immediately implied a parallel (i.e., head-to-head) arrangement of the *At*phyA homodimer, similar to the shape previously observed in cryo-EM studies on *Dr*BphP (Li et al., 2010) and *At*phyB (Burgie et al., 2017). Even prior to more detailed modeling, the lower and upper parts of the density map can be assigned to the PCM and CTM, respectively. Notably, the middle region of the map exhibited a hole of 60 Å × 50 Å in its middle, reminiscent of observations made for *Dr*BphP (Li et al., 2010). Interestingly, the overall strength of the electron density is weaker around the hole, as illustrated by contouring the map at different levels (Figure 4C). Additional, more diffuse density surrounds an ordered core with strong density, in particular near the bottom (assigned to the PCM) and top (assigned to the CTM) of the map (Figure 4C). We tentatively ascribed these density differences to varying degrees of static and dynamic structural heterogeneity in the PCM and CTM. In support of this notion, we note that sensor histidine kinases, and by that token the HKRD in *At*phyA, exhibit inherently high levels of structural malleability that underpins their function (Gushchin and Gordeliy, 2018; Buschiazzo and Trajtenberg, 2019; Möglich, 2019; Multamäki et al., 2020).

**Figure 5:**
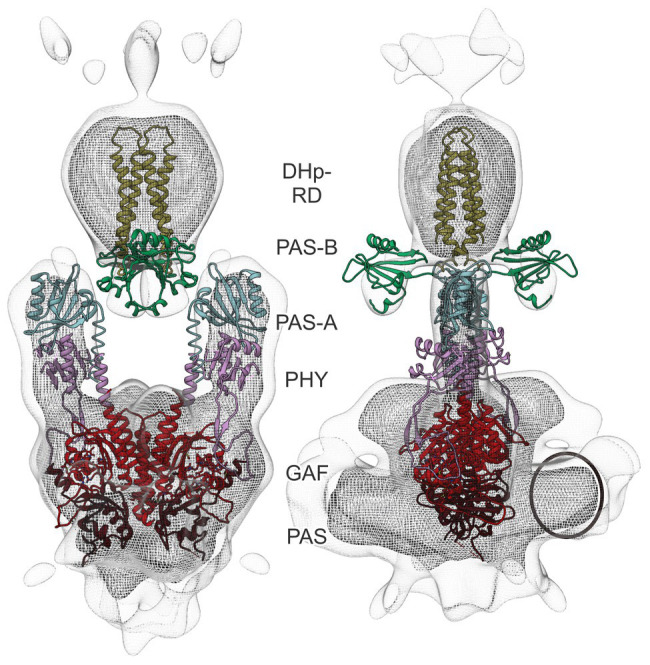
Docking of PCM and CTM domains into the cryo-EM electron density map of *At*phyA. The Per-ARNT-Sim (PAS), cyclic GMP, adenylyl cyclase, FhlA (GAF), and phytochrome-specific (PHY) domains of the PCM are shown in brown, red, and pink, respectively. Additional density in the bottom part of the map (circle) can be ascribed to the NTE. Within the CTM, the PAS-A, PAS-B, and DHp-RD domains are displayed in cyan, green, and yellow, respectively. For details of the modeling, see main text.

To obtain detailed insight into the *At*phyA architecture, we docked structures of the isolated PCM and individual CTM domains (PAS-A, PAS-B, and HKRD) inside the electron density (Figure 5). The PAS-GAF fragment of *G. max* phyA (PDB identifier 6TC7; Nagano et al., 2020) was first positioned in the bottom half of the electron density (Figure 5). To locate the PHY domains, the *At*phyB PCM structure (4OUR; Burgie et al., 2014) was then superposed on the previously placed *G. max* PAS-GAF fragment. Each of the two monomers was placed separately and resulted in a good fit of the electron density for the PHY domains. The model thus indicated that contacts between the two PCM protomers are mainly formed by the PAS and GAF domains, whereas the PHY domains are separated. Notably, this arrangement is supported by ample structural data on bacterial phytochromes across which the position of the PHY domains and the distance between them are quite variable (Takala et al., 2020). The map also revealed additional electron density near the N-terminus of the PCM which can be ascribed to the N-terminal extension (Figure 5, circle). Although no high-resolution structural template is available for the NTE, the spatial location of the surplus density lends credence to our modeling of the PCM.

To model the CTM, a PAS-A fragment was derived from the full-length structure of the *X. campestris* BphP (5AKP; Otero et al., 2016) and placed adjacent to the PHY domain, again resulting in a good fit of the electron density. Next, we jointly positioned the PAS-B and the DHp-RD subdomain of the HKRD using as a model the PAS-linked histidine kinase ThkA from *T. maritima* (3A0R; Yamada et al., 2009). Whereas the DHp-RD antiparallel four-helix bundle well-fitted the top part of the strong electron density, the PAS-B domains were splayed apart and accounted for the weaker electron density below the top of the model (see Figure 4B, lower contour level). Although the PAS-B domains well-fitted the density, we deem their placement tentative given the available data quality. By contrast, the CA-RD subdomain of the HKRD could not be positioned in the electron density with confidence, arguably reflecting its mobility with respect to the DHp-RD module, as evidenced by widely different DHp:CA orientations across sensor histidine kinase structures (Marina et al., 2005; Diensthuber et al., 2013; Wang et al., 2013; Trajtenberg et al., 2016; Buschiazzo and Trajtenberg, 2019).

## Discussion

Notwithstanding the early discovery of plant phytochromes and the important physiological roles they exert, their structural and mechanistic characterization remains incomplete. Although atomically resolved information has become available for several isolated PCMs, the structures of the NTE, the CTM, and the composite photoreceptor have proven elusive. As a corollary, the molecular bases of phytochrome function remain unresolved to significant extent. For instance, although the NTE has been implicated in light-dependent conformational changes and interactions with the phytochrome-interacting factors (von Horsten et al., 2016), the molecular underpinnings of these processes are unclear. Against this backdrop, we set out to obtain structural information on *A. thaliana* phyA at full length by cryo-electron microscopy. A key prerequisite – but common bottleneck – for structural and biophysical analyses is the availability of samples of sufficient quantity and homogeneity. To this end, we established the heterologous expression in *E. coli* of *At*phyA with around 1 mg yield per liter of culture medium. Ligation with the phycocyanobilin chromophore was aided by coexpression of the heme-oxygenase and PcyA enzymes (Mukougawa et al., 2006). Initial attempts at preparing *At*phyA were plagued by aggregation and thus failed to provide monodisperse protein samples. pH variation yielded optimized conditions at which *At*phyA retains photochemical activity and can be isolated as an enriched homodimeric preparation.

Thus equipped, we set up grids with the *At*phyA full-length protein and recorded electron micrographs. Single particles were then picked and analyzed, and the three-dimensional electron density was calculated. The resultant map of about 17 Å resolution revealed an oblong shape of approximately 100 Å × 150 Å × 250 Å size, consistent with a head-to-head homodimeric *At*phyA molecule. Remarkably, the electron density exhibited a hole of 60 Å × 50 Å in its middle. Overall, the principal shape of the electron density and the structural model are strikingly similar to those presented for the *D. radiodurans* BphP (Li et al., 2010). Notably, the hole in the middle of the density has larger dimensions in *At*phyA which we attribute to the PHY and PAS-A domains in the two monomers being separated. By contrast, in the *Dr*BphP model, the PHY domains are paired and the hole results from the linker segment between the GAF and PHY domains of the PCM. As another difference, the *At*phyA map exhibited additional electron density adjacent to the PCM which is accounted for by the NTE. Structural data on full-length bacterial phys indicate that like *Dr*BphP, these receptors predominantly assemble into head-to-head parallel homodimers (Gourinchas et al., 2019; Takala et al., 2020), although other arrangements may be functionally relevant in certain BphPs (Bellini and Papiz, 2012; Otero et al., 2021). Taken together, our analysis indicates that *At*phyA adopts a structure that resembles in key aspects that of the bacterial phytochromes, exemplified by *Dr*BphP (Li et al., 2010; Björling et al., 2016). This structural similarity hints at potential commonalities in signal mechanism between bacterial and plant phytochromes.

Our structural data and the model for *At*phyA are generally compatible with the parallel homodimeric arrangement observed for full-length *At*phyB *via* negative-staining electron microscopy (Burgie et al., 2017). By contrast, the present electron density maps and the structural modeling are not consistent with the cruciform shapes earlier proposed for *At*phyB and for phyA from *P. sativum* (Nakasako et al., 1990, 2005; Oide et al., 2020). The underlying reasons for this discrepancy are unclear but might be found in the respective experimental conditions and differences among plant phytochromes. Further structural investigation of these important plant photoreceptors is clearly warranted. Not least, future studies should be directed at resolving light-induced conformational changes in the entire photoreceptor, that is, beyond the PCM for which pertinent structural data are available, at least in case of bacterial phytochromes. Likewise, cryo-EM analyses seem well-positioned to shed light on the interplay of plant phytochromes with their interacting factors, an area of utmost biological significance yet little molecularly resolved information. To this end, our present work has now established protocols for the preparation of full-length *At*phyA and its analysis by cryo-electron microscopy. By expanding these studies and analyzing more particles in the future, electron density maps with higher resolution will be obtained, potentially allowing to resolve conformational substates and structural heterogeneity.

## Data Availability Statement

The raw data supporting the conclusions of this article will be made available by the authors, without undue reservation.

## Author Contributions

DG cloned, expressed, purified, and analyzed the *At*phyA spectroscopically and chromatographically, and assisted in cryo-EM data collection. WW analyzed the *At*phyA chromatographically, and collected and evaluated the cryo-EM data. SW and AM conceived and supervised the research. All authors interpreted the results. WW and AM wrote the manuscript with input from all authors. All authors contributed to the article and approved the submitted version.

### Conflict of Interest

The authors declare that the research was conducted in the absence of any commercial or financial relationships that could be construed as a potential conflict of interest.
